# ACE2 in the Era of SARS-CoV-2: Controversies and Novel Perspectives

**DOI:** 10.3389/fmolb.2020.588618

**Published:** 2020-09-30

**Authors:** Federica Saponaro, Grazia Rutigliano, Simona Sestito, Lavinia Bandini, Barbara Storti, Ranieri Bizzarri, Riccardo Zucchi

**Affiliations:** ^1^Department of Pathology, University of Pisa, Pisa, Italy; ^2^Department of Chemistry and Pharmacy, University of Sassari, Sassari, Italy; ^3^NEST, Scuola Normale Superiore and CNR-NANO, Pisa, Italy

**Keywords:** angiotensin-converting enzyme 2, ACE2 receptor, cardioprotection, COVID-19, SARS-CoV-2, spike protein, cytokine storm

## Abstract

Angiotensin-converting enzyme 2 (ACE2) is related to ACE but turned out to counteract several pathophysiological actions of ACE. ACE2 exerts antihypertensive and cardioprotective effects and reduces lung inflammation. *ACE2* is subjected to extensive transcriptional and post-transcriptional modulation by epigenetic mechanisms and microRNAs. Also, ACE2 expression is regulated post-translationally by glycosylation, phosphorylation, and shedding from the plasma membrane. ACE2 protein is ubiquitous across mammalian tissues, prominently in the cardiovascular system, kidney, and intestine. ACE2 expression in the respiratory tract is of particular interest, in light of the discovery that ACE2 serves as the initial cellular target of severe acute respiratory syndrome (SARS)-coronaviruses, including the recent SARS-CoV2, responsible of the COronaVIrus Disease 2019 (COVID-19). Since the onset of the COVID-19 pandemic, an intense effort has been made to elucidate the biochemical determinants of SARS-CoV2-ACE2 interaction. It has been determined that SARS-CoV2 engages with ACE2 through its spike (S) protein, which consists of two subunits: S1, that mediates binding to the host receptor; S2, that induces fusion of the viral envelope with the host cell membrane and delivery of the viral genome. Owing to the role of ACE2 in SARS-CoV2 pathogenicity, it has been speculated that medical conditions, i.e., hypertension, and/or drugs, i.e., ACE inhibitors and angiotensin receptor blockers, known to influence ACE2 density could alter the fate of SARS-CoV-2 infection. The debate is still open and will only be solved when results of properly designed experimental and clinical investigations will be made public. An interesting observation is, however that, upon infection, ACE2 activity is reduced either by downregulation or by shedding. These events might precipitate the so-called “cytokine storm” that characterizes the most severe COVID-19 forms. As evidence accumulates, ACE2 appears a druggable target in the attempt to limit virus entry and replication. Strategies aimed at blocking ACE2 with antibodies, small molecules or peptides, or at neutralizing the virus by competitive binding with exogenously administered ACE2, are currently under investigations. In this review, we will present an overview of the state-of-the-art knowledge on ACE2 biochemistry and pathophysiology, outlining open issues in the context of COVID-19 disease and potential experimental and clinical developments.

## Introduction

Angiotensin-converting enzyme (ACE) has an important role in the metabolism of several peptides and proteins, including chemical messengers, such as angiotensin I and bradykinin. The importance assumed by research focused on ACE biochemistry, physiology and pharmacology is closely linked to the clinical effectiveness showed by ACE inhibitors in the treatment of cardiovascular disease, particularly arterial hypertension and congestive heart failure. At present, ACE inhibitors are among the most widely prescribed drugs worldwide.

In 2000 a novel ACE-like enzyme, eventually named ACE2, was discovered. It showed a different substrate selectively, and in particular it could not activate angiotensin I. At the same time, it was not a target of classical ACE inhibitors. Investigations were therefore started, aiming at determining the role of ACE2, and the general hypothesis that ACE2 may counteract several physiological consequences of ACE activation was introduced. In 2003 ACE2 was also identified as the initial cellular target of the SARS-CoV virus, since its spike glycoprotein was found to be a high affinity ligand of membrane ACE2. Similar properties are shared by SARS-CoV-2 virus, and the huge impact of the recent COVID-19 pandemic has triggered novel interest in ACE2, particularly in the regulation of its expression in different cell types.

Hypertension has been suggested to have a major impact on COVID-19 susceptibility and prognosis, therefore the elusive link between hypertension, anti-hypertensive drugs, and ACE2 expression has soon become the object of a major controversy. On the other hand, treatments able to interfere with ACE2 expression, or ACE2 availability for SARS-CoV-2 binding, have been identified as a novel goal of pharmaceutical research.

The purpose of the present review is to summarize the available knowledge on ACE2 biochemistry and biology. We have also tried to point out some crucial open questions, which should be addressed to provide a better background for future experimental and clinical investigations.

## ACE System and ACE2 Discovery

Human Angiotensin-converting enzyme (ACE) belongs to the M2 gluzincin family of metalloproteinases and (Ehlers and Riordan, 1989; Masuyer et al., 2014) exists in two forms, namely somatic ACE (sACE) and germinal ACE (tACE). Both are derived from the same gene, controlled by alternative promotors. sACE is an integral membrane protein, which can be also cleaved by ACE secretases to produce a circulating form of the enzyme (Natesh et al., 2003).

sACE, hereafter referred to simply as ACE, has been extensively studied, because of its crucial role in the homeostasis of renin-angiotensin-aldosterone (RAAS) system and in cardiovascular diseases (Takimoto-Ohnishi and Murakami, 2019). The two extracellular domains N and C domains of ACE (Wei et al., 1991; Jaspard et al., 1993; Natesh et al., 2003; Riordan, 2003) can both hydrolase two crucial peptides, namely angiotensin I and bradykinin, with the same efficiency. Indeed, ACE carries out the cleavage of two amino acids (dipeptidase action) from the C-terminal part of angiotensin I to generate angiotensin II, which exerts a potent vasopressor, proliferative, and profibrotic effect. Moreover, ACE mediates the cleavage and inactivation of bradykinin, which is a vasodilator hypotensive peptide. The pivotal role of ACE in the RAAS system allows a refined blood pressure control and salt homeostasis (Sayer and Bhat, 2014).

Following the ACE discovery in mid-1950s, despite intense research in the field, no human homologs of the enzyme have been found for more than 50 years (Isaac et al., 1998; Riordan, 2003). It was only in 2000 that two independent research groups identified, almost simultaneously, a new human ACE-like enzyme, with two different approaches. Tipnis et al. (2000) searched for new metalloproteases in an expressed sequence tag (EST) database, finding an ACE homolog (ACEH) with a single domain, similar to that of insects. Subsequently they cloned it from a human lymphoma cDNA library. Interestingly ACEH showed high homology (40% identity and 60% similarity) with ACE, particularly around the HEXXH sequence and highly conserved glutamate residue, involved in zinc binding. Moreover, they demonstrated the presence of seven glycosylation sites (Tipnis et al., 2000). In the same year, Donoghue et al. (2000b) were searching for new genes involved in heart failure and identified a human cDNA ACE homolog, named ACE2, among 19,000 5’end sequences by RACE (rapid amplification of cDNA ends) in the heart ventricle cDNA library, obtained from a woman with idiopathic dilated cardiomyopathy. ACE2 showed a transmembrane domain, a zinc catalytic domain 42% identical to ACE and a signal peptide. Like ACE, ACE2 seemed to be an ectoenzyme type I protein. The authors identified ACE2 transcripts quite exclusively in the heart and in the kidney, suggesting a role for ACE2 in the local RAAS control (Donoghue et al., 2000b). In the following years, ACE2 was intensively studied, its structure and function were enlightened, and tentative inhibitors were developed.

## ACE2: Structure, Expression, Tissue Distribution, and Function

### Structure of ACE2

*ACE2* is a 40 kb gene and it is positioned on chromosome Xp22, differently from *ACE* gene that is located on chromosome 17. The 18 exons of *ACE2* are remarkably similar to *ACE* exons. The *ACE2* gene depicts a large polymorphism and several novel polymorphisms of *ACE2*, with specific geographical distribution, have been described and associated with susceptibility to hypertension and cardiovascular disease (Burrell et al., 2013; Patel et al., 2014; Pinheiro et al., 2019).

The *ACE2* gene codifies for a typical zinc-metallopeptidase of 805 amino acids (120 kDa), with a unique catalytic domain. Despite the high resemblance of ACE and ACE2, considerable differences exist in their substrates and products. While ACE acts as dipeptidase, ACE2 removes only a single amino acid from its substrates. Therefore, ACE2 is not active in transforming angiotensin I to angiotensin II and in inactivating bradykinin; moreover, ACE2 is insensitive to ACE inhibitors, like lisinopril and captopril (Tipnis et al., 2000; Rice et al., 2004; Turner et al., 2004).

**FIGURE 1:**
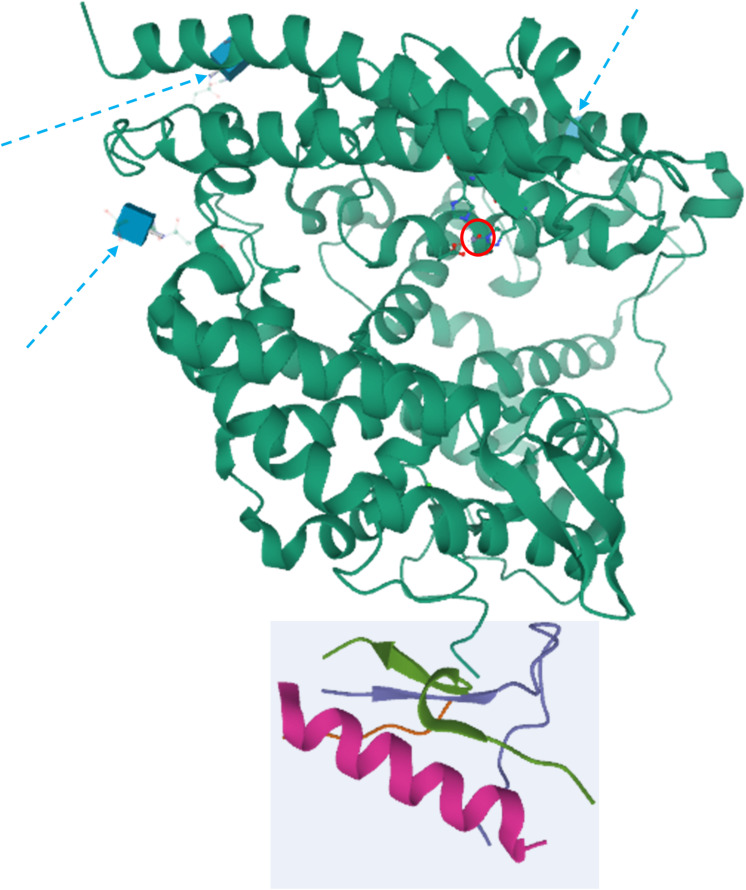
Crystal structure of ACE2. The peptidase domain (PD) is in green, whereas the collectrin homology domain is enclosed in the light cyan square. The active zinc ion is showed enclosed in a red circle, whereas the glycosylation moieties are showed as cyan cubes and denoted by dashed arrows. The structures have been drawn from PDB 1R42 (Towler et al., 2004) by Mol on the PDB website.

These differences depend on variances in the three-dimensional structure (3D) of the two enzymes. Comparative homology modeling and crystallography contributed to shed light on ACE2 3D structure (Figure 1). Prabakaran et al. (2004) clarified the major characteristic of ACE2, which is a deep channel on the summit of the protein, hosting the catalytic domain. Specific loops, like the long loop N210-Q221 that is exclusive of ACE2, α helices and a portion of β-sheet are located around the catalytic channel. The negative charge of the channel and the presence of distinct hydrophobic regions contribute to the specificity of the binding site (Prabakaran et al., 2004). The determination of the crystal structure of the extracellular domain to 2.2-3-A resolution from Towler et al. (2004) and the model from Guy et al. (2003) showed that the catalytic domain of ACE and ACE2 are very conserved and have similar mechanisms of action. The main difference stems from the smaller ACE2 pocket, thereby lodging only a single amino acid: the crucial substitution of the Gln281 in ACE binding pocket with Arg273 in ACE2 is likely to be responsible for the steric conflict (Guy et al., 2003; Towler et al., 2004). Another surprise of the ACE2 structure was its C-terminal domain, which—differently from ACE—revealed high homology with collectrin, a renal protein, which is involved in amino acids trafficking through the membrane (Yang et al., 2017).

### Transcriptional, Post-transcriptional, and Post-translational Regulation of ACE2

The location of the *ACE2* gene on the X chromosome questions whether one of the two *ACE2* is silenced in females, to balance female/male expression dosage (X chromosome inactivation or XCI), or otherwise belongs to the class of “escape genes” which are transcribed on both chromosomes. Interestingly, a wide survey of XCI in several individuals and tissues showed that *ACE2* is a heterogeneous escape gene, because it has a tissue-dependent sex bias (Tukiainen et al., 2017).

A growing number of recent findings point to an important role of epigenetic mechanisms associated with several human diseases (Surguchov et al., 2017). In this context, several authors highlighted the regulatory role of 17β-estradiol (E_2_), a primary female sex steroid, in the expression of *ACE2* in a tissue-dependent fashion. Liu et al. (2010) and Stelzig et al. (2020) found out that E_2_ downregulates *ACE2* in kidney and differentiated airway epithelial cells, respectively. The latter result is particularly important, as the male-bias of *ACE2* expression in the lung could account for the alleged higher susceptibility of males to COVID-19 symptoms following ACE2-dependent SARS-CoV-2 infection (section Links Between ACE2 and COVID-19) (Jin et al., 2020). Yet, Bukowska et al. (2017) observed that E_2_ increases *ACE2* transcription and expression in human atrial tissue, while at the same time depressing the level of ACE protein. This mechanism attenuates the renin-angiotensin system and, in tandem with anti-inflammatory and anti-oxidative effects, enables a stronger response to myocardial stress and contributes to antiarhythmic effects. The upregulation of *ACE2* (and downregulation of *ACE*) was clearly linked to binding of E_2_ to Estrogen Receptor alpha (ERα) (Bukowska et al., 2017). The E_2_-ERα complex might migrate to the nucleus to bind to estrogen response elements, although the actual mechanism is still obscure and should likely involve other co-factors to take into account the observed tissue bias of E_2_ regulation. Nonetheless, it was demonstrated that the Estrogen Related Receptor alpha (ERRα), which likewise ERα recognizes the estrogen response element in target genes, binds to *ACE2* promoter to repress transcription (Tremblay et al., 2010; Lee et al., 2012). Hopefully, future studies will shed more light on the intriguing role of estrogens in *ACE2* regulation.

A recent analysis of public genomic and transcriptomic data outlined the role of histone methylation, a classical epigenetic mark, to regulate *ACE2* transcription. Indeed, Li Y. et al. (2020) showed that transcription of *ACE2* was significantly upregulated when the histone mutant H3K27M was overexpressed to inhibit H3K27me3. Conversely, overexpression of mutant H3K4/9/36M did not change *ACE2* transcription. Trimethylation of K27 on H3 is catalyzed by the polycomb groups (PcG), a group of conserved transcriptional gene repressors (Schuettengruber et al., 2017). PcG proteins assemble into two major complexes: PRC1 and PRC2. The simplest model of PcG activity involves trimethylation of H3 by PRC2 at target gene promoters (Blackledge et al., 2015). These epigenetic marks recruit PRC1 on DNA, which in turn acts as E3-ligase and ubiquitinates nearby H2A histones (Storti et al., 2019), triggering silencing of gene transcription by local and reversible compaction of chromatin (Illingworth, 2019). The catalytic subunit of PRC2 is constituted by the EZH2 protein. In agreement with the inverse correlation between *ACE2* level and H3K27me3, *ACE2* expression in human ESCs was upregulated following EZH2 knock-out (Li Y. et al., 2020). On the other side, recovery of EZH2 restored basal *ACE2* levels. Chromatin immunoprecipitation sequencing (ChIP-seq) showed that EZH2 depletion induced H3K27me3 decrease, with concomitant H3K27ac increase, at *ACE2* promoter in human ESCs (Li Y. et al., 2020). The role of H3 methylation and acetylation in the epigenetic regulation of ACE2 was also hypothesized by Pinto et al., who demonstrated that co-morbidities such as hypertension, diabetes, and chronic obstructive lung disease increase *ACE2* transcription in the lung (Pinto et al., 2020).

Histone methylation does not appear to exhaust the epigenetic regulation of ACE2. Notably, the NAD^+^-dependent deacetylase SIRT1 binds to *ACE2* promoter favoring its transcription during cellular energy stress (Clarke et al., 2014). Two recent unrefereed preprints highlighted other epigenetic mechanisms at play. Corley et al.^1^ pointed out that DNA methylation across three CpG islands in the *ACE2* promoter was lower in lung epithelial cells compared to other cell types, suggesting high transcription in lung tissue. These findings are in excellent agreement with the reported inverse correlation between *ACE2* transcription and promoter methylation in tumors, which will be discussed in section ACE2 and Other Diseases. This correlation is also supported by the observation that in children *ACE2* is normally hypermethylated and poorly expressed either in the lung and in other organs (Pruimboom, 2020). Glinsky (2020) addressed the epigenetic role of Vitamin D on *ACE2* expression, showing by gene set enrichment analysis that the Vitamin D receptor (VDR) should be involved in a set of regulatory pathways conveying on *ACE2*. More specifically, VDR activation would downregulate *ACE2*, thus affording a potential reason for the alleged beneficial role of Vitamin D in COVID-19 (section ACE2 and the Inflammatory Response to Sars-CoV-2).

Finally, the strict homeostatic balance of ACE/ACE2 activities suggests transcriptional co-regulation of both proteins. Remarkably, Yang et al. (2016) have demonstrated that a subtle regulatory mechanism acts in cardiac endothelial cells, where the Brg1 chromatin remodeler and the FoxM1 transcription factor cooperate to determine the ACE2/ACE expression ratio, particularly under cardiac hypertrophy of the heart.

Further regulation occurs at the mRNA level. From putative microRNA-binding sites identified *in vitro*, Lambert et al. (2014) demonstrated that miR-421 downregulates ACE2. According to the hypothesized mechanism, miR-421 modulates ACE2 expression by hampering translation rather than by degradation of mRNA transcripts.

Beside undergoing post-translational modifications by glycosylation and phosphorylation, ACE2 is also post-translationally regulated by shedding from cell membrane through the action of the metalloproteinase ADAM17. The proteolysis of ACE2 releases a soluble, enzymatically active form which corresponds to the ACE2 ectodomain (Jia et al., 2009; Xiao et al., 2014; Conrad et al., 2016). The function, if any, of soluble ACE2 is still obscure, but the shedding mechanism is under strict molecular control. Lambert et al. (2008) highlighted that calmodulin associates with ACE2 to prevent its shedding, while calmodulin inhibitors increase the cellular release of ACE2. Patel identified a positive feedback in the RAAS: Ang II activates ADAM17, thereby increasing the release of ACE2, its negative regulator (Patel et al., 2016b). It is worth noting that high levels of plasma-soluble ACE2 have been associated with myocardial dysfunction (Epelman et al., 2009). The potential pathophysiological role of ADAM17 is further discussed in paragraph ACE2 and the Inflammatory Response to Sars-CoV-2.

**FIGURE 2:**
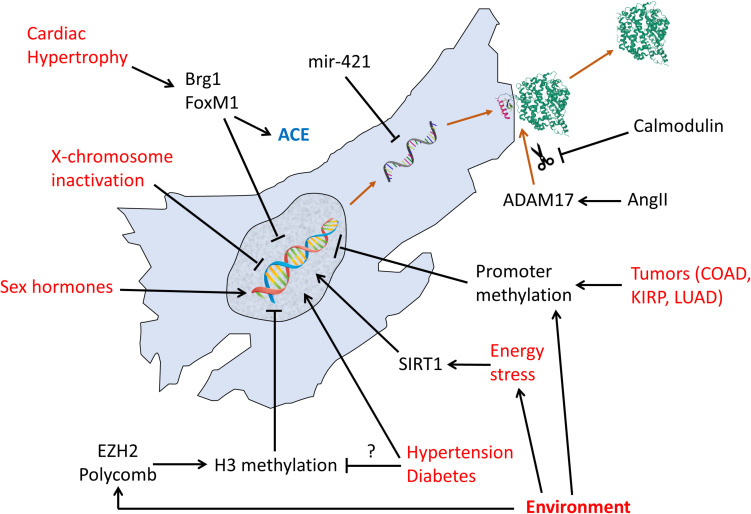
Regulation pathways of transcription, translation, and post-translational shedding of ACE2. Red text: non-molecular factors. Black text: molecular factors.

Figure 2 summarizes the known transcriptional, post-transcriptional, and post translational regulation pathways of ACE2.

### Tissue Distribution of ACE2

Detectable quantities of ACE2 protein have been found almost ubiquitously in tissues across mammalian species, using immunostaining methods. ACE2 is predominantly located in the cardiovascular system and kidney, where it probably plays a role in the maintenance of hydro electrolyte homeostasis (section “Mechanism of Viral Entry Mediated by the S Protein”). In fact, ACE2 is pervasively expressed throughout the vasculature, at the level of the arteries and veins, mainly in smooth muscle cells of the media and in the endothelium (Hamming et al., 2004; Burrell et al., 2005). Such signal from vessels also delivers part of the expression detected in specific organs. Indeed, ACE2 is evident in: coronary vessels and myocardial capillaries (Wiener et al., 2007; Garabelli et al., 2008); lung microvascular endothelial cells (Wiener et al., 2007; Chen et al., 2013); kidney interlobular arteries (Lely et al., 2004); endothelial and smooth muscle cells in the brain (Hamming et al., 2004; Kar et al., 2010). Notably, the mesangium and glomerular endothelium in the kidney, and the endothelial lining of the sinusoids in the liver are allegedly negative for ACE2 (Hamming et al., 2004). On the contrary, ACE2 is virtually absent from the lymphatic system, and human hemato-lymphoid organs (i.e., spleen, lymph nodes, and bone marrow) (Hamming et al., 2004; Li et al., 2007). In blood cells, it has been observed in platelets and macrophages, but not in B and T lymphocytes (Hamming et al., 2004; Fraga-Silva et al., 2011).

Expression of ACE2 was originally identified in rodent heart (Donoghue et al., 2000a), where it was observed to occur in both atrium an ventricle (Gembardt et al., 2005), and, cellularly, in cardiomyocytes and in specialized cells of the sinoatrial node (Burrell et al., 2005; Garabelli et al., 2008; Ferreira et al., 2011; Wang et al., 2017). In human heart, ACE2 has been found in the stromal area in spongiosa layer in aortic valves (Peltonen et al., 2011), where it is expressed in myofibroblasts and fibroblasts (Guy et al., 2008).

High levels of ACE2 protein expression have been detected in mammalian, including human, kidney (Gembardt et al., 2005; Koka et al., 2008; Reich et al., 2008; Giani et al., 2012; Mitani et al., 2014; Grobe et al., 2015; Shi et al., 2015; Larouche-Lebel et al., 2019; Alawi et al., 2020). Strong signals were reported in the brush border of the proximal tubular cells, whereas weak to moderate signals could be found in the glomeruli, Henle’s loop, distal tubules, and collecting duct (Hamming et al., 2004; Lely et al., 2004; Kamilic et al., 2010; Giani et al., 2012; Bae et al., 2015; Cao et al., 2017; Errarte et al., 2017).

In the respiratory tract of primates, positive labeling for ACE2 has been reported at multiple sites, from the nasal and oral mucosa, to the larynx, trachea, bronchi and lung (Liu et al., 2011). Whether ACE2 is expressed in human nasal and oral epithelium remains unclear, as contradictory results have been reported by studies using immunohistochemistry (Hamming et al., 2004; Bertram et al., 2012), in face of positive single-cell RNA sequencing findings (Sungnak et al., 2020). This point is of great interest to understand the role of those tissues in SARS-CoV-2 initial infection, spread and clearance (section “Links Between ACE2 and COVID-19”). In the upper respiratory tract ACE2 is expressed in the epithelial lining and lamina propria, in some muscle cells and in the salivary gland duct epithelium. In the lung, an intense signal for ACE2 protein has been consistently observed in type I and II pneumocytes in several species, including mouse, rat, cat, ferret, monkey and human (Wiener et al., 2007; van den Brand et al., 2008; Liu et al., 2011; Wong et al., 2012; Chen et al., 2013; Zhang B. N. et al., 2019). Data from rodents suggest an age- and gender-dependent pattern of expression, with a more rapid decline with age in males as compared to females (Xie et al., 2006).

Although some ACE2 signal has been observed in the liver, it appears to mainly come from small vessel endothelium, and occasionally bile duct epithelial cells, while negligible expression is observed in hepatocytes (Hamming et al., 2004; Paizis et al., 2005; Guan et al., 2020). ACE2 protein is abundantly expressed in the brush border of enterocytes of all parts of the small intestine, including the duodenum, jejunum, and ileum, but not in enterocytes of the colon. Other organs of the digestive tract, such as the stomach and colon, did not show brush border staining, but rather a positive signal in the muscolaris mucosae and the muscolaris propria (Hamming et al., 2004). In rodents, ACE2 is also expressed in both exocrine and endocrine pancreatic tissue, particularly in the islets of Langerhans (Niu et al., 2008; Fang and Yang, 2010).

ACE2 distribution is widespread in the mouse brain, from the telencephalon to the medulla. As expected, ACE2 is found in brain areas involved in the regulation of cardiovascular function and fluid balance, such as the vascular organ of lamina terminalis, subfornical organ, magnocellular neurons in the hypothalamic paraventricular nucleus, area postrema, nucleus of the solitary tract, dorsal motor nucleus of the vagus, nucleus ambiguous, and rostral ventrolateral medulla (Doobay et al., 2007). However, significant expression had also been reported in brain areas not engaged in the classical functions of the RAAS, namely the piriform cortex, hippocampus, caudate putamen, hypoglossal nucleus and primary motor cortex (Doobay et al., 2007; Lin et al., 2008; Liu et al., 2014). ACE2 immunostaining was identified in neurons as well as astrocytes (Gallagher et al., 2006; Doobay et al., 2007; Yamazato et al., 2007). Furthermore, ACE2 has been documented in the retina, predominantly in the inner nuclear layer but also in photoreceptors (Tikellis et al., 2004; Senanayake et al., 2007).

With regard to the endocrine system, ACE2 expression was found in both male and female reproductive systems. In human testis, ACE2 was localized to the Leydig and Sertoli cells, and might be involved in testicular function (Douglas et al., 2004). At present, no data about ACE2 protein expression is human ovaries is available, although evidence of expression in stroma, theca, and granulosa cells has been reported in other species (Tonellotto dos Santos et al., 2012; Barreta et al., 2015; Pereira et al., 2015).

In rodent bone, ACE2 is expressed in osteoblasts and osteoclasts, as well as in epithelial cells and fibroblasts. However, a similar expression in human samples still awaits clarification (Queiroz-Junior et al., 2019).

In human skin, ACE2 was present in the basal cell layer of the epidermis extending to the basal cell layer of hair follicles. Smooth muscle cells surrounding the sebaceous glands were also positive for ACE2. Weak cytoplasmic staining was observed in sebaceous gland cells. A strong granular staining pattern for ACE2 was seen in cells of the eccrine glands. Positive staining for ACE2 was also noted in the membrane of human fat cells in various organs, including the epicardial adipose tissue (Hamming et al., 2004; Patel et al., 2016a). Globally, ACE2 is chiefly bound to cell membranes, while negligible levels can be detected in the circulation.

### ACE2, the RAAS System and Cardioprotection

After the initial discovery of ACE2 in the heart and kidney, it is now clear that it is widely distributed in tissues (section Tissue Distribution of ACE2), where it exerts many physiological effects and may be involved in pathophysiological events (Turner, 2015). The effect of ACE2 which has been more extensively investigated is the regulation of the RAAS system, where ACE2 counter-balances ACE, limiting the potent vasoconstrictive effect of angiotensin II (Ang-II). The first evidence that ACE2 was involved in RAAS control came from the transgenic knockout mouse model (ACE2^–/–^), which was characterized by severe reduction of cardiac contractility and thinning of the left ventricular wall. Interestingly, in this knockout model disruption of the ACE pathway could rescue the myocardial phenotype (Crackower et al., 2002). In another study, a selective ACE2 knockout model showed high blood pressure, worsened by the infusion of Ang-II (Gurley et al., 2006).

**FIGURE 3:**
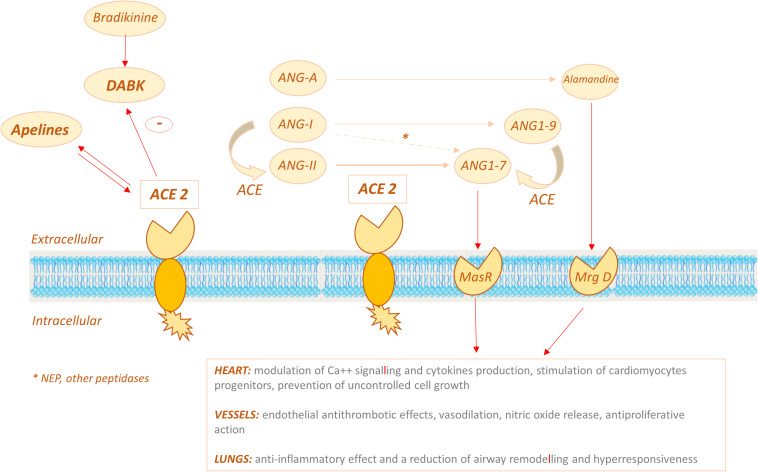
ACE2 signalling pathways: ACE2 displays its carboxypeptidase activity converting Ang-II (ANG II) to ANG1–7 and can also convert angiotensin I (ANG-I) to the nonapeptide Ang1–9, which is in turn converted into ANG1–7 by ACE. ANG1-7 binds the MasR receptor to exert its effects on target organs (primarily heart, vessels, lungs). ACE2 might also act via the bradykinin-DABK/BKB1R axis: the increased activity of the DABK axis triggers a proinflammatory cytokine storm.

As a matter of fact, ACE2 displays its carboxypeptidase activity converting Ang-II to a heptapeptide, namely Ang1–7 (Turner et al., 2004). ACE2 can also convert angiotensin I (Ang-I) to the non apeptide Ang1–9, which is in turn converted into Ang1–7 by ACE, competing with Ang-I and thus further decreasing Ang-II (Arendse et al., 2019; Figure 3). Ang1–7 has been demonstrated to bind to the MasR receptor, which was initially regarded as an orphan receptor, since the use of a MasR antagonist caused inhibition of Ang1–7 effects (Alenina et al., 2008).

In the last years the ACE2/Ang1–7/MasR system has been intensively studied: physiological effects on cardiomyocytes include modulation of Ca^++^ signaling and cytokine production, stimulation of cardiomyocytes progenitors and prevention of uncontrolled cell growth (Grobe et al., 2006; Flores-Muñoz et al., 2012; Souza et al., 2013; Chang et al., 2016). Through the Ang1–7 pathway ACE2 produces endothelial antithrombotic effects, vasodilation, nitric oxide release, and inhibits vascular smooth muscle cells (VSMC) proliferation (Loot et al., 2002; Sampaio et al., 2007a,b; Fraga-Silva et al., 2008). In preclinical studies Ang1–7 displayed antifibrotic effects, protecting from deleterious myocardial hypertrophy and modulating left ventricle remodeling after myocardial infarction (MI). In animal models, Ang1–7 also showed an antiarrhythmic action (Ferreira et al., 2001; Santos et al., 2004; Grobe et al., 2006; Gomes et al., 2010), while compensatory ACE2 upregulation has been observed in explanted human hearts, in patients affected by ischemic or dilated cardiomyopathy (Goulter et al., 2004; Burrell et al., 2005).

Additional players contribute to ACE2-mediated cardio-protection. Another substrate of ACE2 carboxypeptidase activity is Angiotensin A (Ang-A), identified in 2008, which has a similar, although less potent, vasopressor effect as AngII (Jankowski et al., 2007). The product of the enzymatic reaction catalyzed by ACE2 is the peptide Ala-Ang (1–7) also known as alamandine (Lautner et al., 2013; Figure 3). Alamandine receptor is a Mas-related G-protein coupled receptor, known as MrgD, which is expressed in cardiomyocytes and blood vessels. MrgD knockout animals develop a severe cardiopathy and alamandine showed a cardioprotective effect in a model of sepsis (Santos et al., 2019).

Unlike ACE, ACE2 is not active on bradykinin, but it can degrade the active bradykinin metabolite des-Arg9 bradykinin (DABK), blocking the signaling pathway of the B1 bradykinin receptor (Vickers et al., 2002; Sodhi et al., 2018).

Other substrates of ACE2 include apelin-13/17, neurotensin, dynorphin A (1–13), and ghrelin (Turner, 2015). Interestingly peptides of apelin family have been demonstrated to upregulate ACE2 expression in physiological condition and more actively during heart failure in *in vivo* experiments (Sato et al., 2013). ACE2 in turn is able to regulate apelin bioavailability, establishing a negative feedback loop, and the crosstalk between RAAS, ACE2 and Apelin system might play a significant role in the pathophysiology of hypertension (Kalea and Batlle, 2010; Chen et al., 2015).

On the whole these findings suggest that ACE2 might exert antihypertensive and/or cardioprotective effects by different mechanisms, namely: (i) limiting the availability of ACE substrates, (ii) degrading Ang-II, (iii) activating the Ang1-7/MasR and/or Alamandine/MrgD pathways, (iv) interfering with other substrates, such as DABK and apelins (Figure 3).

### ACE2 and Lung Injury

ACE2 is highly expressed in type I and II alveolar epithelial cells and in pulmonary small vessels (either endothelial cells or VSMC). The hypothesis that the ACE2 arm of the RAAS system could be of benefit in lung disease derives from the observation that ACE and angiotensin II are upregulated in acute lung injury (ALI), pulmonary fibrosis, pulmonary hypertension, and acute respiratory distress syndrome (ARDS) (Imai et al., 2005, 2010).

ARDS is an overly aggressive form of ALI and it is the final mechanism of lung injury in many diseases, including sepsis, acid aspiration, pancreatitis, anthrax and virus infections (Spanish flu, H5N1 avian flu and SARS). Imai et al. developed three *ACE2* knockout mice models with severe ARDS induced by acid aspiration, endotoxin administration or peritoneal sepsis. They showed that ARDS was accompanied by increased vessel permeability, lung oedema, and infiltration of inflammation cells, with consequent impairment of respiratory function (Imai et al., 2005, 2007; Kuba et al., 2006). The phenotype was dramatically improved and rescued either administering ACE2 recombinant analogs or AT1Ra inhibitors (Imai et al., 2005, 2008).

ACE2 may also reduce lung inflammation via Ang1–7/MasR, since treatment with recombinant Ang1–7 in a model of allergic asthma reduced eosinophil mobilization, peri-bronchial inflammation, fibrosis and goblet cells metaplasia (El-Hashim et al., 2012). An anti-inflammatory effect with reduction of airway remodeling has also been demonstrated in another model of chronic asthma, after administration of Ang1–7 analog (Rodrigues-Machado et al., 2013). The underlying mechanism seems to be the modulation of the so-called cytokine storm and particularly the inhibition of transforming growth factor β (TGF- β) and NFkB signaling pathways (Li et al., 2015; Meng et al., 2015).

ACE2 might also modulate lung inflammation via the bradykinin-DABK/BKB1R axis. In fact, decreased ACE2 function in mouse lungs caused increased activity of the DABK axis and triggered a proinflammatory cytokine storm (CXCL5, MIP2, KC, and TNF-α), leading to pulmonary collapse (Sodhi et al., 2018).

In human, ACE/ACE2 imbalance may be related to genetic factors. In particular, a specific polymorphism of the *ACE* gene, namely *ACE D*, which determines increased ACE activity and decreased ACE2 activity has been correlated to ARDS susceptibility and mortality rate (Marshall et al., 2002). More recently, high levels of angiotensin II have been reported in patients infected with avian influenza viruses H5N1 and H7N9, and they were strongly predictive of a poor outcome (Huang et al., 2014; Zou et al., 2014).

## Links Between ACE2 and COVID-19

A “second life” for ACE2 was discovered in 2003, when a novel respiratory infective disease, known as severe acute respiratory syndrome (SARS), appeared in China and spread all over Asia and Canada, with a lethality rate of 10% (Rota et al., 2003). The responsible pathogen was identified in a positive strand RNA virus belonging to the coronavirus family and named SARS-CoV (Ksiazek et al., 2003). The virus genome was sequenced and this allowed the identification of the spike glycoprotein (S), whose N-terminal portion, or S1-domain, was found to mediate the high affinity binding to host cells (Marra et al., 2003). The group of Farzan et al. succeeded in identifying the cell receptor: they showed that SARS-CoV efficiently infected African Monkey kidney cell line Vero E6 and co-immunoprecipitated a glycoprotein responsible for virus binding and entry, which was identified as ACE2 (Li et al., 2003).

SARS-CoV caused the SARS epidemic in 2002–2003, with over 8,000 infections and a fatality rate around 10%. In late 2019, another coronavirus emerged as a human pathogen in the city of Wuhan in China, producing symptoms such as fever, severe respiratory impairment, and pneumonia (Petersen et al., 2020). This new coronavirus has been denominated SARS-CoV-2 for its genetic resemblance with SARS-CoV (∼80%), and its related disease has been named COVID-19 (COronaVIrus Disease 2019). Owing to its high reproduction number R_0_ (2–3), SARS-CoV-2 has rapidly diffused in several countries and is currently posing a significant global health threat. On March 11, 2020, the WHO has declared the COVID-19 outbreak a pandemic. The early discovery that SARS-CoV-2 also engages ACE2 as entry door for cell infection has prompted an intense research effort to elucidate the biochemical determinants of CoV-ACE2 interactions.

### Mechanism of Viral Entry Mediated by the S Protein

**FIGURE 4:**
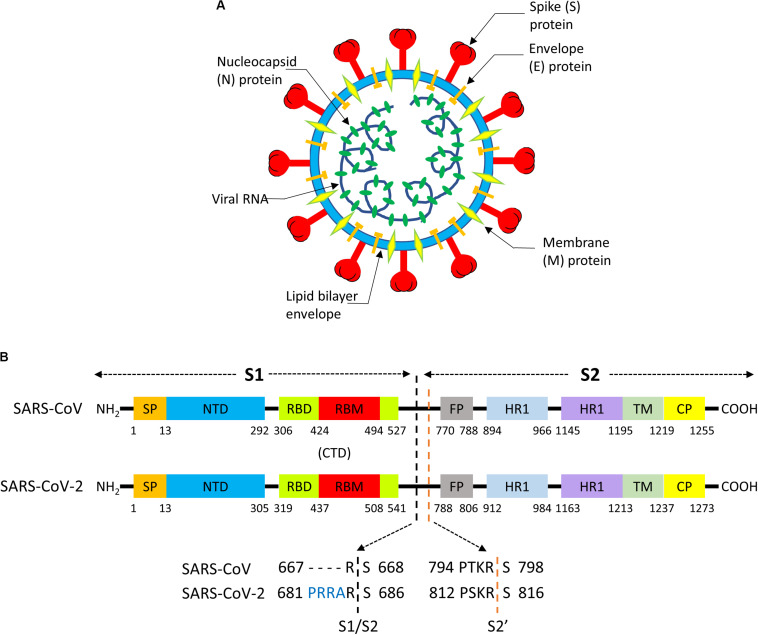
**(A)** Structure of a Coronavirus. **(B)** Functional motifs in the sequence of the S “spike” protein of SARS-CoV and SARS-CoV-2.

A coronavirus contains four structural proteins, namely spike (S), envelope (E), membrane (M), and nucleocapsid (N) proteins. These proteins assemble around a lipid bilayer to provide the shell enclosing the viral genome (Figure 4A; Tang et al., 2020). Homotrimers of S protrude from the viral surface, and are densely decorated by N-linked glycans, creating the “crown” (“Corona” in Latin) that christens this virus group (Walls et al., 2016). S is a ∼180 kDa glycoprotein anchored in the viral membrane, which plays the most important roles in viral attachment, fusion and entry (Ou et al., 2020). Sequence analysis has shown that SARS-CoV-2 S protein shares 76% of the primary sequence with the corresponding S of human SARS-CoV (Ou et al., 2020). Accordingly, it has been early proposed that SARS-CoV-2 utilizes a similar cell entry mechanism as SARS-CoV, pivoted on S protein. This hypothesis has been confirmed from an experimental point of view. By using pseudotyped virus bearing SARS-CoV S or SARS-CoV-2 S, it was shown that a large panel of cell lines allows comparable entry of SARS-CoV or SARS-CoV-2 viruses (Hoffmann et al., 2020b; Walls et al., 2020).

The S protein consists of ∼1300 aminoacids and it is composed by a N-terminal “S1”subunit (∼700 aa) and a C-terminal “S2”subunit (∼600 aa); binding to the host receptor is mediated by S1, whereas S2 induces fusion of the viral envelope with cellular membranes (Walls et al., 2017). S1 and S2 can be further subdivided in functional segments with different roles in viral entry (Figure 4B; Tang et al., 2020). The S1 subunit contains two subdomains, the N-terminal domain (NTD) and the C-terminal domain (CTD). In SARS-CoV (Li, 2015) and SARS-CoV-2 (Wang et al., 2020) CTD encloses the receptor-binding domain (RBD), and the RBD section that directly contacts the receptor is named as receptor-binding motif (RBM). The N-region of S2 contains a fusion peptide (FP), two heptapeptide repeat domains (HR1, HR2), a transmembrane domain (TM), and cytoplasmic peptide (CP). FP is a short segment composed of mostly hydrophobic residues, such as glycine (G) or alanine (A), which inserts in the host cell membrane to trigger the fusion event. HR1 and HR2 are composed of a repetitive heptapeptide with HPPHCPC sequence, where H represents hydrophobic or bulky residues, P polar or hydrophilic residues, and C charged residues. HR regions typically fold into α-helices with a hydrophobic interface that drives membrane fusion.

On the basis of the strong similarity between the S proteins of SARS-CoV and SARS-CoV-2, many researchers early set-out to demonstrate whether both viruses target the same host cell receptor, ACE2. Zhou et al. (2020) highlighted that the virus was able to infect cell lines only when they expressed human, bat, civet, and pig (but not mouse) ACE2 receptor. Hoffmann et al. (2020b), Ou et al. (2020), and Walls et al. (2020) elegantly outlined that the BHK cell line could be infected by pseudotyped SARS-CoV-2 or SARS-CoV only upon ACE2 expression. Conversely, the expression of different human receptors used by other CoVs (hDPP4 and APN, used by MERS CoV and HCoV-229E, respectively) did not enable pseudovirus access to cells (Hoffmann et al., 2020b). Taken together, these findings are solid evidence that SARS-CoV-2 engages ACE2 for cell entry.

Nonetheless, the two viruses were demonstrated by Xia to share also the membrane fusion mechanism, as strongly suggested by the impressive 89.9% sequence identity of S2 between SARS-CoV and SARS-CoV-2 (Xia et al., 2020a,b).

**FIGURE 5:**
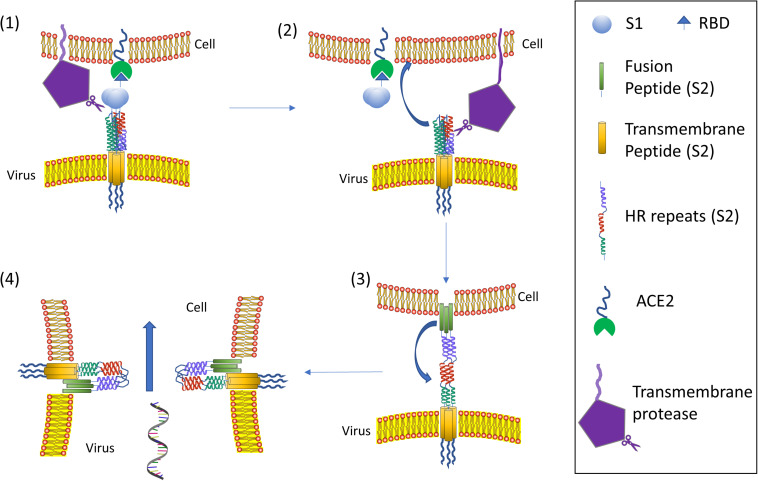
Coronavirus viral fusion pathway model. Initially, the S protein is in the pre-fusion native state (1). Then S undergoes priming of the S1 subunit at S1/S2 by local proteases yielding the pre-fusion metastable state (2); note that priming at S1/S2 could also happen upon virus formation in releasing cell: in such a case the virus attaches to a host cell already in the pre-fusion metastable state (2). Subsequent triggering by a protease on S2’ enables the FP to insert in the host membrane upon the “jack-knife” transition of HR1 and HR2 yielding the pre-hairpin intermediate (3). The pre-hairpin folds back on itself due to HR1 and HR2 interactions eventually forming the post-fusion (6) state. During the S protein foldback, the two membranes approach each other until the outer leaflets merge (hemifusion) and eventually the inner leaflets merge (pore formation). Note that cell membrane may refer to plasma membrane (direct fusion) or endosomal membrane (fusion in endocytic vesicle). Adapted from Tang et al. (2020).

**FIGURE 6:**
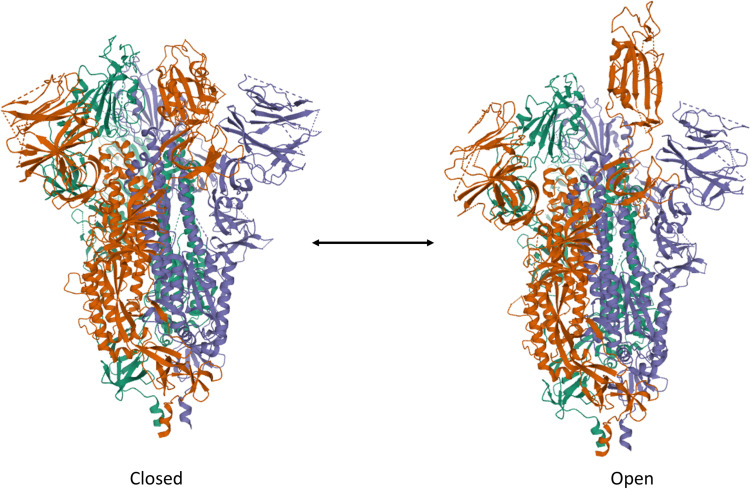
Trimeric S protein of SARS-CoV-2 in the **”**Closed” and “Open” forms. Note the single RBD protruding out of the V-shaped conformation of the protein assembly. The structures have been drawn from PDB 6X2C (R. Henderson, 10.1101/2020.05.18.10208) by Mol on the PDB website.

To date, the cell entry mechanism of SARS-CoV and SARS-CoV-2 has been understood in its general details and it is based on a concerted action of receptor binding and proteolysis of the S protein (Figure 5; Tang et al., 2020). Ultrastructural studies showed a metastable “prefusion” V-shaped trimer composed by three S1 heads sitting on top of a trimeric S2 stalk anchored into the virus membrane (Walls et al., 2016). The RBD constantly switches between a standing-up (“open”) position for receptor binding and a lying-down (“closed”) configuration, the latter allowing immune evasion (Figure 6; Song et al., 2018; Wrapp et al., 2020). Yet only one of the three RBD in trimeric S can flip up at a time and interact with the receptor (Wrapp et al., 2020). The second key feature of the fusion mechanism is “priming” by host proteases, which recognize and cleave a short peptide motif at the S1/S2 boundary (Figure 4B). This cleavage does not disassemble S1 from S2 in pre-fusion conditions (Belouzard et al., 2009), but the binding interaction of RBD with its receptor, accompanied by a further cleavage in a second site in S2 (S2’site, upstream of FP, Figure 4B), triggers the possible dissociation of S1 and the irreversible refolding of S2 into a “post-fusion” state (Figure 4B). In detail, HR1 undergoes a dramatic “jack-knife” conformational change, converting four helical stretches that run in an antiparallel fashion into a single long (∼130 aa) α-helix (Heald-Sargent and Gallagher, 2012; Walls et al., 2017). At first, three of these helices assemble into a homotrimeric bundle and stick the FP into the host cell membrane. Then, HR2 (one for each S2 chain) fold backward and bind to HR1, yielding the “six-helix bundle fusion core” (6-HB) of post-fusion S2 (Song et al., 2018). This conformational foldback brings the FP (at N-terminus of HR1) and the TM (at the C terminus of HR2) close to each other, so that the viral and host cell membranes approach until their outer leaflets merge (hemifusion, Figure 5). Eventually the inner leaflets merge (pore formation), enabling a connection between the interior of the virus and the host cell cytoplasm, that allows the delivery of viral genome (Figure 5; Tang et al., 2020).

**FIGURE 7:**
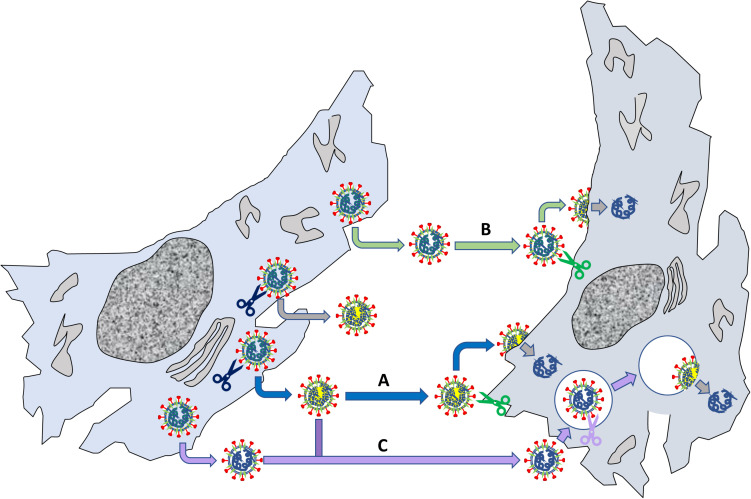
Relevance of S S1/S2 “priming” by host proteases for viral fusion to cells. The left cells produce viruses that can be “primed” by endogenous proteases such as furin (blue scissors); other viruses are not primed when they exit the cell. The primed viruses (marked by a yellow internal shadow) reach another cell (pathway A), where a membrane protease (e.g., TMPRSS2) may cleave the S2’ site (see Figure RB1b) leading to membrane fusion and delivery of viral RNA. Non-primed viruses can deliver their genome by two routes: in B, the virus reaches the cell, is primed on the membrane at both S1/S2 and S2’ by a local protease and then fuse with the plasma membrane; alternatively, in C the virus is internalized by endocytosis and priming/fusion occurs in endocytic vesicles. Note that also “primed” viruses may undergo pathway C, depending on their interaction with the recipient cell.

Although not directly related to ACE2, the role of S “priming” by host cell proteases deserves particular attention in the context of SARS-CoV-2 virus entry and tropism. Possibly, the most notable feature of SARS-CoV-2 genome, as compared to SARS-CoV and some related bat coronaviruses, is a four basic aminoacid insert (PRRA) at the S1/S2 junction (Figure 4B; Jaimes et al., 2020). This site is potentially cleavable by the protease furin, a proprotein convertase widely recognized to activate the fusion machinery of viral glycoprotein. Indeed, many authors showed that pseudoviruses bearing SARS-CoV-2 S were already “primed” (i.e., cleaved) at the S1/S2 boundary by furin upon assembly in the cell, at odds with pseudoviruses bearing SARS-CoV S (Shang et al., 2020a). SARS-COV-2 shows a large flexibility with regard to protease priming, which may independently occur by a) furin and furin-like proteases intracellularly, b) trypsin-like proteases such as TMPRSS2 that are present on the host cell membrane (particularly on airway epithelial cells), and 3) endosomal cathepsins activated by a drop in pH (e.g., cathepsin L) (Figure 7; Hoffmann et al., 2020a,b). This flexibility could be the crucial factor that explain SARS-CoV-2 cell tropism and the peculiar features of COVID-19 symptoms (Jaimes et al., 2020). Additionally, the kind of protease “priming” may determine whether the membrane fusion process occur directly at the plasma membrane or at endosomal level (Tang et al., 2020; Figure 7).

### SARS-CoV-2 RBD and Its Receptor, ACE2

Since SARS-CoV-2 and SARS-CoV share the same host cell receptor, it was early questioned whether SARS-CoV-2 retains the same RBD motif of SARS-CoV. SARS-CoV RBD corresponds to residues 306–527 of S protein (Li et al., 2005). Sequence analysis shows that residues 319–541 of SARS-CoV-2 (S_319__–__341_) share 73.9% sequence identity with SARS-CoV RBD (Wang et al., 2020). Accordingly, Wang et al. (2020) clearly demonstrated that S_319__–__341_ corresponds to SARS-CoV-2 RBD by immunofluorescence microscopy. Indeed SARS-CoV-2 S1 and S_319__–__341_ positively colocalized with GFP-tagged ACE2 expressed on cell surface of HEK cells, whereas this interaction did not occur with membrane-expressed hDPP4 (MERS receptor). Additionally, soluble ACE2 inhibited the interaction between viral proteins and ACE2-expressing cells in a dose-dependent manner.

**FIGURE 8:**
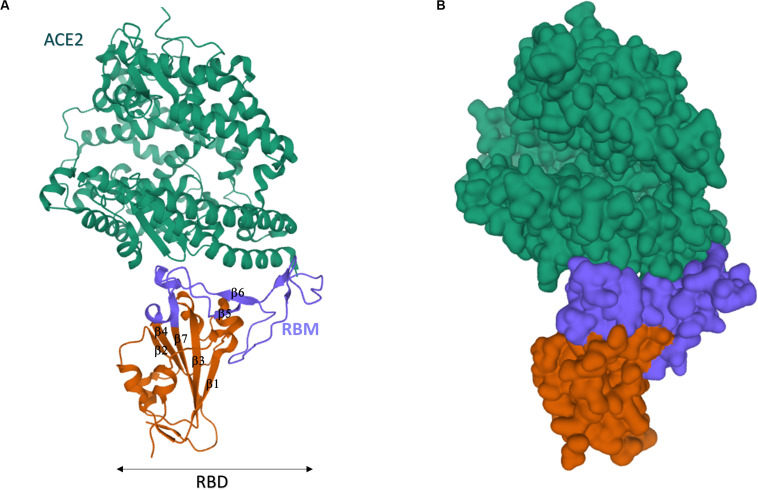
Crystal structure of SARS-CoV-2 spike receptor-binding domain bound with ACE2. **(A)** Cartoon representation. **(B)** Gaussian surface representation. hACE2 is in green, the core of SARS-CoV-2 RBD is in red, and the RBM is in blue. The β1-β7 typical motifs of RBD (Lan et al., 2020) are indicated in **(A)**. The structures have been drawn from PDB 6MOJ (Lan et al., 2020) by Mol on the PDB website.

The SARS-CoV-2 RBD sequence was further investigated by structural analysis. X-ray crystallography showed that SARS-CoV-2 RBD folds into two structural domains (Figure 8): (1) the core subdomain with five antiparallel β-strands (β1, β2, β3, β4, β7), (2) the external subdomain, which inserts between β4 and β7, and it is characterized by the two small β5 and β6 strands [β1’ and β2’ in Wang et al. (2020)] connected by a disulfide bond (Lan et al., 2020; Wang et al., 2020). In keeping with their high sequence homology, the 3D structure of SARS-COV-2 and SARS-CoV RBD nearly superimpose (RMSD = 0.475 Å for 128 Cα atoms; Wang et al., 2020) with the exception of the β5/β6 loop, which actually entailed the larger primary sequence difference.

Several researchers investigated the interaction of SARS-CoV-2 RBD with ACE2. Unfortunately, each group committed to slightly different sequences of SARS-CoV-2 RBD. To avoid confusion, we will always report the actual sequence with respect to the S protein when the RBD under study differs from the canonical 319–541 stretch. *In vitro* affinity studies revealed dissociation constants of the ACE2-RBD complex in the 1–100 nM range (Table 1). Non univocal data are attributable to the dissimilar sequences that were investigated and/or to the immobilization procedures (Shang et al., 2020b). In spite of this variability, SARS-CoV-2 RBD was always found to bind ACE2 4–10 fold stronger than SARS-CoV RBD (Lan et al., 2020; Shang et al., 2020b; Walls et al., 2020; Wang et al., 2020). The affinity difference *in vitro* was confirmed also *in vivo* by the stronger binding of SARS-CoV-2 S_331__–__524_ to ACE2 expressed on cells (SARS-CoV-2: EC_50_ = 0.08 μg/ml *vs.* SARS-CoV: EC_50_ = 0.96 μg/ml) (Tai et al., 2020). Paradoxically, however, it has been shown that the ACE2 binding affinity for the entire SARS-CoV-2 S protein is lower than or comparable to that of SARS S (Shang et al., 2020a). This surprising result suggests that SARS-CoV-2 RBD, albeit more potent, is less efficiently exposed than SARS-CoV RBD by the dynamic transition between the “closed” and “open” states, probably in order to escape the immune system of the host. Thus, the non-identical S1 sequences of SARS-CoV-2 and SARS-CoV reflect the molecular evolution of SARS-CoV-2 toward: (1) much stronger affinity toward ACE2, (2) reduced antigenicity of the RBD region (which is one of the most antigenic segments in the S protein), (3) greater and less specific cleavability by host proteases. Taken together, these properties account for the sophisticated strategy exploited by SARS-CoV-2 to invade host cells.

**TABLE 1:** Binding affinity between SARS-CoV-2 spike (S) protein and S subset regions and ACE2.

Sequence	K_*D*_ (nM)	Method	References
SARS-2 S	14.7	SPR	Wrapp et al., 2020
SARS-2 S	11.2	SPR	Lei et al., 2020
SARS-2 S_20__–__685_ (S_1_)	94.6	SPR	Wang et al., 2020
SARS-2 S_319__–__541_ (RBD)	133.3	SPR	Wang et al., 2020
SARS-2 S_319__–__541_ (RBD)	4.7	SPR	Lan et al., 2020
SARS-2 S_319__–__529_	44.2	SPR	Shang et al., 2020b
SARS-2 S_319__–__591_	34.6	Biolayer interferometry	Wrapp et al., 2020
SARS-2 S_328__–__533_	1.2	Biolayer interferometry	Walls et al., 2020

### Structure of the ACE2-RBD Complex

Previous studies showed that the extracellular Peptidase Domain (PD) of ACE2, which adopts a claw-like morphology, is the interaction site of SARS-CoV RBD (Towler et al., 2004; Li, 2015; Kirchdoerfer et al., 2018; Song et al., 2018). More specifically, ACE2 engages SARS-CoV RBD by establishing contacts with the 424–494 residue domain, which is referred to as Receptor Binding Motif (RBM) (Li et al., 2005).

In spite of the recent emergence of SARS-CoV-2, multiple authors have already highlighted the structure of ACE2/SARS-CoV-2 RBD complex by either X-ray (Lan et al., 2020; Shang et al., 2020b; Wang et al., 2020) or cryo-EM (Walls et al., 2020; Wrapp et al., 2020; Yan et al., 2020). Pleasantly, all data converged to a consistent tridimensional arrangement of the receptor (Wang et al., 2020). In the “closed” conformation of S protein, the RBD is buried at the interface between protomers (Walls et al., 2020). Only in the “open” S conformation, RBD engages PD of ACE2 (Wrapp et al., 2020), and the complex may involve a dimeric ACE2 that accommodates two S protein trimers (Yan et al., 2020). In keeping with their sequence similarity, strong structural homology was found between ACE2/SARS-CoV RBD and ACE2/SARS-CoV-2 RBD (Lan et al., 2020; Shang et al., 2020b; Wang et al., 2020). SARS-CoV-2 RBM spans from residue 438–506 of S sequence and, likewise SARS-CoV RBM, it approaches the outer surface of ACE2 by a gently concave surface with a ridge on one side (Figure 8). The concave surface is made up by the two short β5 and β6 sheets of the external RBD subdomain, whereas the ridge contains the β5/β6 loop (loop 1: residues 474–489). A second smaller loop (loop 2: residues 498–505) is visible on the other side of the concave surface. Inspection of the complex structure and molecular dynamics (MD) highlighted that the motifs 453–456 (in β5), 484–489 (in the loop 1), and 500–505 (in the loop 2) are at the basis of the largest differences between SARS-CoV-2 and SARS-CoV RBM interactions with ACE2. SARS-CoV RBM ridge contains a Pro-Pro-Ala motif that is replaced by Gly-Val-Glu-Gly in SARS-CoV-2 (residues 482–485), yielding a more compact loop able to engage more interactions with proximal ACE2 residues (e.g., Ser^19^ and Gln^24^) (Shang et al., 2020b). Additionally, Phe^486^ of SARS-CoV-2 RBM (which replaces Ile of SARS) inserts into a hydrophobic pocket on the receptor surface, establishing strong aromatic interactions with Tyr^83^ of ACE2 (Wang et al., 2020). Asn^501^ in loop 2 further engages recognized hotspots on the ACE2 surface (Shang et al., 2020b). Consistently, MD studies confirmed that loop 1 and 2 are much more rigid in RBM-ACE2 complex of SARS-CoV-2 with respect to SARS (Brielle et al., 2020). These subtle structural differences probably account for the higher affinity of SARS-CoV-2 for ACE2 (Wang et al., 2020). Interestingly, MD simulations suggest that the difference in affinity is largely due to the solvation energy, emphasizing the relevant role of hydrophobic patches in RBM/ACE2 binding surface (He et al., 2020).

It is worth noting that the RBD-receptor engagement is the crucial effector of viral-host interaction, which eventually determines viral host range, and in tandem with the host proteases is responsible for virus tropism and pathogenicity (Millet and Whittaker, 2015). Structure-guided sequence analysis has suggested that several mammals, including pets such as cats and dogs, host ACE2 receptors that could bind effectively to SARS-CoV-2 S protein and propagate COVID-19 infection (Luan et al., 2020). Yet, no correlation between genetic distance and the S/ACE2 interaction was found.

In this context, a third human CoV, hCoV-NL63, has been previously found to use ACE2 for cell entry (Hofmann et al., 2005), although its S1 sequence is rather dissimilar from SARS (23.4%) and SARS-CoV-2 (29.2%). In spite of this, the structures of hCoV-NL63 (Wu et al., 2009) and SARS-CoV RBD (Li et al., 2005) were found to engage some sterically overlapping sites in ACE (Li, 2015). This homology can be transitively extended to SARS-CoV-2, suggesting these three CoVs have evolved to recognize a “hotspot” region in ACE2 for receptor binding. This might represent a critical feature for the appearance of novel CoV able to infect humans in the future, as it is known that at least three more bat CoVs bind ACE2 (Hoffmann et al., 2020b). Indeed, bat RaTG13 CoV binds ACE2 and contains a similar four-residue motif in the ACE2 binding ridge of RBM, suggesting that SARS-CoV-2 could have evolved from RaTG13 or a yet-unknown related bat CoV (Shang et al., 2020b).

### The Controversial Role of ACE2 Expression

Epidemiological data consistently show that the COVID-19 patients at highest risk of a poor prognosis are males older than 60 years with chronic underlying diseases, mostly hypertension, cardiovascular diseases and type-2 diabetes mellitus. Clinical reports have been rapidly delivered from all over the world, and meta-analyses assessing the prevalence of comorbidities and their impact on prognosis are already available. A meta-analysis pooling data from seven studies following a total number of 1,576 infected patients from hospitals in China found that the most prevalent comorbidities were hypertension (21.1%), diabetes (9.7%), and cardiovascular diseases (8.4%). These increased the risk of developing a more serious disease (i.e., requiring intensive care treatment), with odds ratios ranging from 2.4 (hypertension) to 3.4 (cardiovascular disease) (Yang et al., 2020b). These findings have been confirmed in the analysis performed by the Chinese Center for Disease Control and Prevention in a huge sample of 72314 COVID-19 cases (Epidemiology Working Group for Ncip Epidemic Response and Chinese Center for Disease Control and Prevention, 2020). A study with 1591 Italian patients, similarly, reported a significant association between hypertension and mortality in Intensive Care Unit (63 vs. 40%). This series reported an even higher prevalence of hypertension (49%), diabetes (17%), and cardiovascular disease (21%) (Grasselli et al., 2020). Diabetes has been reported to predict the occurrence of ARDS (HR = 1.44), acute kidney injury (HR = 3.01), septic shock (HR = 1.95), and all-cause mortality (HR = 1.70) (Zhu et al., 2020). Notably, poor glycemic control was significantly associated with worse clinical outcomes, namely multi-organ injuries and higher mortality (Zhu et al., 2020). Obesity has also emerged as an important factor in determining COVID-19 severity. Indeed, obesity was more frequent in patients admitted to critical care for SARS-CoV-2 as compared to the general population; moreover, the BMI was positively related to the need for invasive mechanical ventilation and mortality (Drucker, 2020; Simonnet et al., 2020).

The RAAS system is the target of widely used anti-hypertensive drugs, such as ACE-inhibitors (ACEI) and Ang-II type 1 receptor blockers (ARB). Several experimental studies reported that, although not directly affecting ACE2 activity, these drugs are able to upregulate its expression (Ishiyama et al., 2004; Ferrario et al., 2005; Gallagher et al., 2008). Increased expression of ACE2 has also been reported to facilitate SARS-CoV infection in several experimental models and postulated to act in the same way for SARS-CoV-2 (Li et al., 2003; Hofmann et al., 2004; Perrotta et al., 2020).

Hence, following the SARS-CoV-2 outbreak, it has been speculated that the use of ACEI and ARB could increase viral invasion and should therefore be temporarily suspended. This topic has been abundantly debated in the last few months (March–May 2020), with contradictory views (Bavishi et al., 2020; Buckley et al., 2020; Danser et al., 2020; Fang et al., 2020; Huang Z. et al., 2020; Kreutz et al., 2020; Kuster et al., 2020; Mourad and Levy, 2020; Park et al., 2020; Rico-Mesa et al., 2020; Sommerstein et al., 2020; South et al., 2020; Tignanelli et al., 2020; Vaduganathan et al., 2020; Verdecchia et al., 2020b; Zhang P. et al., 2020). As a consequence of this debate, several clinical societies have stated that suspension of ACEI and ARB is not justified on the basis of the present scientific evidence, although a recent BMJ editorial (Aronson and Ferner, 2020) suggested to consider stopping ACE inhibitors or angiotensin receptor blockers in patients with mild hypertension who are at high risk of coronavirus infection.

On the other hand, a different viewpoint is based on the intriguing observation that several conditions increasing the risk of viral infection and disease severity are all characterized by a certain degree of ACE2 deficiency. As discussed above (section “Structure of ACE2”) ACE2 deficiency has been suggested to play a significant role in the pathophysiology of hypertension, cardiovascular disease and diabetes. The role of ACE2 expression in the panel of comorbidities correlated to SARS-CoV-2 is a matter of intense still unsolved debate, as brilliantly revised by Shyh et al. (2020) in a very recent paper.

On the whole, there is still uncertainty about the relationship between ACE2 density on cell membrane and the fate of SARS-CoV-2 infection. An interesting speculation which could reconcile the two position comes from the hypothesis that while ACE2 is for sure the entry door for the virus, once the infection has evolved, there is the subsequent downregulation of ACE2, responsible for the precipitating of respiratory distress, as showed also in animal models (Kuba et al., 2005).

It should be pointed out that the great clinical impact of this issue has encouraged discussions that are largely based on indirect speculations rather than objective data. Data from appropriate clinical trials are still lacking, although a retrospective multicenter study on more than 1,000 patients with COVID-19 and hypertension treated with ACEI or ARB revealed that the use of these drugs was associated with lower mortality from all cause (Bosso et al., 2020).

A definite conclusion would require specific prospective clinical trials, which are still running: https://clinicaltrials.gov/ct2/show/NCT04312009 is a multi-centered double blind trial with the aim to test the role of ARB in patients with COVID-19 and https://clinicaltrials.gov/ct2/show/NCT04331574 is another trial evaluating the outcome of patients with COVID-19 in therapy with either ACEI and ARBS.

In the meanwhile, partial but important information might be obtained by circumscribed experimental and clinical investigations focused some on crucial issues, namely: fate of SARS-CoV-2 infection in human cells manipulated in order to modify ACE2 density; correlation of ACE2 density (in different cell types) and clinical course in human patients; potential role of additional proteins interacting with ACE2, such as the membrane transporter SIT1, which appears to be associated with COVID-19 prognosis and is also affected by anti-hypertensive therapy.

### ACE2 and the Inflammatory Response to SARS-CoV-2

Differently from other flu-like viral infections, COVID19 showed a relatively high lethality, although the latter was quite different in different geographical areas. The appearance of ARDS and acute severe lung injury was initially regarded as the main insult, and patients underwent the typical treatment developed for ARDS. However, more recent evidence from autopsy series revealed that COVID-19 is a systemic disease, in which interstitial pneumonia is associated with extensive microvascular damage, which is not limited to the lungs and leads to fibrin deposition, neutrophils trapping in microvasculature, and arteriolar microthrombi. Moreover, cardiovascular involvement has been observed, occasionally leading to myocardial injury and sudden death after weeks from the initial infection. These findings are crucial to optimize the treatment of COVID-19 patients, hopefully improving the prognosis (Buja et al., 2020; Carsana et al., 2020; Magro et al., 2020).

Another determinant of the clinical picture and a negative prognostic factor is an aggressive proinflammatory response of the host to the infection. Huang described the clinical features of 41 patients admitted to their Institute in January of 2020 with confirmed COVID-19 and pneumonia. In 29% (*n* = 12) of patients ARDS occurred and in 10% invasive mechanical ventilation was required. These patients clearly showed increased production of IL-1β, IFN-γ, IP-10, MCP-1, IL-4, and IL-10 compared to healthy people. Moreover, the patients who required access to the intensive care unit had significantly higher levels of proinflammatory cytokines compared to those who did not require intensive care, suggesting that this “cytokine storm” may trigger the development of the most severe forms of the disease (Huang C. et al., 2020).

**FIGURE 9:**
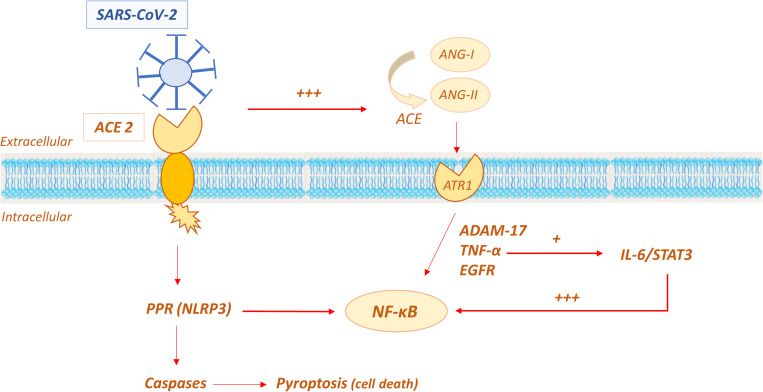
Possible role of ACE2 in the molecular mechanisms involved in the “cytokine storm” of SARS-CoV-2.

The molecular mechanisms involved in the cytokine storm and the role of ACE2 are currently still not fully understood. As discussed above, virus binding to the ACE2 is the event that initiates viral replication in susceptible cells, such as alveolar epithelial cells, vascular cells and immune system cells (macrophages, monocytes). This has been suggested to trigger the primary inflammatory response, which involves apoptosis and pyroptosis (Fu et al., 2020). The apoptosis pattern indices cell death to avoid viral replication in the absence of overt inflammation, whereas, pyroptosis is a violent form of programmed cell death, followed by an inflammatory storm (Cookson and Brennan, 2001; Liu et al., 2016). In the standard pyroptosis model, when the pathogen enters the host cell, some specific structures on the pathogen surface (PAMPs–pathogen associated molecular patterns) are identified by pattern recognition receptors (PRRs) on the host membrane. One common PRR is NOD-like receptors protein 3 (NLRP3), which forms together with a protein caspase activating protein (ASC or apoptosis-associated speck-like protein containing a caspase recruitment domain) and pro-caspase 1, the inflammasome unit capable of recruiting proinflammatory cytokines and inducing cell lysis with further inflammation signals (Fernandes-Alnemri et al., 2007; Schroder and Tschopp, 2010; Wree et al., 2014; Figure 9).

In the SARS-CoV-2 infection, pyroptosis has been demonstrated to be active in macrophages, being viroporin 3a a trigger for NLRP3 with subsequent production of proinflammatory cytokines, such as IL-1β (Chen et al., 2019). In COVID-19 patients, the pyroptosis activation has been indirectly demonstrated by the high serum level of IL-1β (Huang C. et al., 2020). Moreover, the severe leucopoenia and lymphopenia, commonly showed in COVID-19 pneumonia and associated with poor prognosis, are likely due to lymphocyte injury by pyroptosis (Yang, 2020).

Another proposed mechanism that triggers the proinflammatory response to SARS-CoV-2 is ACE/ACE2 imbalance in the RAAS system. It has been speculated that when ACE2 is occupied by the virus, its effects in limiting the amount and activity of Ang-II is decreased; Ang-II is consequently increased and it has been reported to induce a proinflammatory effect via the AT1R receptor (Marchesi et al., 2008; Eguchi et al., 2018). The Ang-II/AT1R system activates disintegrin and the metalloprotease ADAM 17 (also known as TNFα cleavage enzyme TACE), which lead to the intracellular production of epidermal growth factor ligands (EGFR) and TNFα production, with subsequent stimulation of the transcription factor NF-K B, a pivotal player in proinflammatory cytokines release. Additionally, ADAM 17 activation induces the production of the soluble form of IL-6Rα, active on IL-6-STAT3 pathway, which in turn amplifies NF-K B signaling (Murakami et al., 2019; Takimoto-Ohnishi and Murakami, 2019; Palau et al., 2020). The convergence on hyperactivation of NF-K B seems to be crucial in inducing the cytokine storm. The mechanism is self-feeding, given that NF-K B induces the expression of the angiotensinogen gene, amplifying the Ang-II inflammatory response (Costanzo et al., 2003; Hirano and Murakami, 2020).

ACE2 shedding is an additional mechanism contributing to inflammation (Fu et al., 2020). ACE2 shedding consists in proteolytic cleavage of the protein ectodomain and leads to the release of enzymatically active soluble ACE2 (sACE), whose action is not completely understood, but might enhance the pro-inflammatory response (Guy et al., 2005). ADAM 17 and disintegrin are able to shed not only TNFα but also a variety of membrane-anchored enzymes, including ACE2 (Black et al., 1997; Palau et al., 2020), as demonstrated by Lambert in 2005 (Guy et al., 2005). In their experiment, human embryonic kidney cells (HEK293) expressing human ACE2 (HEK-ACE2) were treated with phorbol myristate acetate stimulation and the production of a polypeptide of 105 kDa was demonstrated by western blot analysis, suggesting the occurrence of a proteolytic shedding in the ectodomain of ACE2. Subsequently, they demonstrated that the cleavage was performed by ADAM17, incubating cells with a specific hydroxamic acid-based metalloproteinase inhibitor, namely TAPI-1 (TNFα protease inhibitor 1) (Guy et al., 2005).

ACE2 shedding seems to be inducible by specific stimuli produced either by a viral infection or by the immune system. Jia et al. (2009) confirmed ACE2 shedding in differentiated primary airway epithelial cells and Calu-3-cell line. Furthermore, they demonstrated that the incubation with IL-1β at the dosage of 100 ng/ml and TNF α 100 ng/ml induced ACE2 shedding and sACE release, with a maximum after 18 h of incubation (Jia et al., 2009). These findings suggest that an inflammatory response is likely to modulate ACE2 expression and shedding, also during a viral infection. Following this data, ADAM17 inhibition was proposed as a possible pharmacological target in SARS-CoV-2, but further studies are needed to evaluate this hypothesis (Palau et al., 2020).

Other conditions have been assumed to be involved in the susceptibility to SARS-CoV-2 and among them vitamin D deficiency was proposed as a credible candidate. The interesting candidate could be identified as a modifiable risk factor (hypovitaminosis D) and a potential tool in COVID-19 prevention or ancillary treatment. The rationale has been summarized by Grant et al. in a recent review on the evidence supporting a possible correlation between vitamin D and SARS-CoV-2 risk: (i) the seasonal flare of SARS-CoV-2 which coincides with the nadir of vitamin D levels, (ii) the association between hypovitaminosis D and pulmonary infections together with the demonstrated protective role in acute respiratory infections, in adults (iii) the anti-inflammatory role of vitamin D which could be of benefit against the so called “cytokine storm,” which seems to be a major player in SARS-CoV-2 morbidity and mortality (Martineau et al., 2017; Zhou et al., 2019; Grant et al., 2020).

### ACE2 as a Therapeutic Target

**FIGURE 10:**
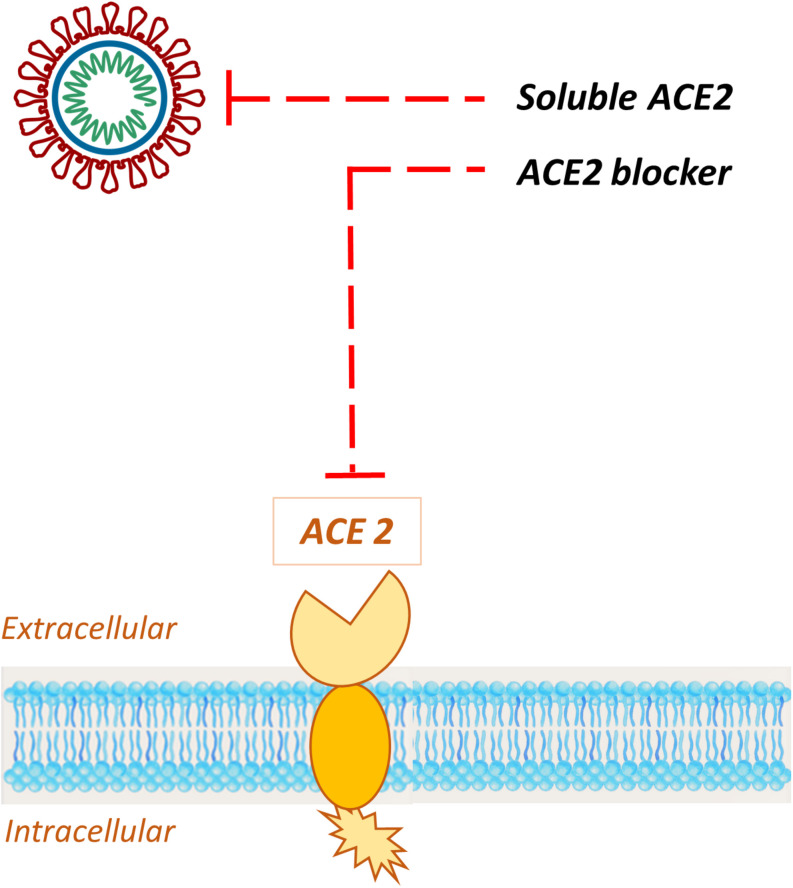
Schematic representation of the potential therapeutic approaches to address ACE2 mediated infection. Exogenous administration of soluble recombinant human ACE 2 sequesters circulating viral particles, while specific ACE2 blockers bind the receptor, impeding the S-protein interaction with the host target.

As evidence builds up, ACE2 rapidly emerged as a specific target for COVID-19 treatment. Since this enzyme was identified as the SARS-CoV-2 receptor (Zhou et al., 2020), several approaches to address ACE2 mediated infection have been described (Li Y. et al., 2020; Zhang H. et al., 2020), with the aim to prevent host cell entry and subsequent viral replication, as well as severe lung injury. Potential therapeutic approaches include (Figure 10):

1. ACE2 blockers;
2. Exogenous administration of ACE2.

#### ACE2 Blockers

Blockade of ACE2 receptor could be achieved through specific antibodies (Li et al., 2003), rationally designed small molecules (Dales et al., 2002; Huentelman et al., 2004; Gross et al., 2020; Pillaiyar et al., 2020) or peptides (Huang et al., 2003). Although their efficacy needs to be confirmed, some of these agents are currently available on the market and have been show to effectively block SARS-CoV invasion (Li S.-R. et al., 2020). For instance, the small synthetic inhibitor N-(2-aminoethyl)-1aziridine-ethanamine (NAAE) binds ACE2 active site in its closed conformation; this contact triggers the shifting of SARS-CoV S binding residues preventing the molecular interaction with targeted enzyme and the subsequent cell-cell fusion (Huentelman et al., 2004). Therefore, although ACE2 catalytic site is distinct from the S-protein-binding domain, NAAE exerts dual inhibitory effects on ACE2 catalytic activity and SARS binding (Adedeji and Sarafianos, 2014). However, since a protecting role for ACE2 receptor against virus-induced acute lung injury in infections with SARS coronavirus has not been excluded (Imai et al., 2005; Kuba et al., 2005; Li S.-R. et al., 2020), the choice of ACE2 inhibition as therapeutic approach should be carefully evaluated.

#### Exogenous Administration of ACE2

The administration of a large amount of soluble form of ACE2 could represent an intriguing opportunity, since excessive ACE2 may exert dual functions: (a) competitively bind SARS-CoV-2 to neutralize the virus and/or slow viral entry in the host cell; (b) rescue cellular ACE2 activity, which negatively regulates RAAS and may theoretically exert a protective effect in lung injury (Verdecchia et al., 2020a). A pilot clinical study is currently investigating the efficiency of a recombinant human angiotensin-converting enzyme 2 (rhACE2) in patients with COVID-19 (Zhang H. et al., 2020).

Recombinant human ACE 2, rhACE2 (hrsACE2, APN01, GSK2586881), sequesters circulating viral particles interfering with S-protein binding to its host target, beside its role in regulating the systemic RAAS. Taken together, these activities may offer therapeutic benefits in COVID-19 patients, although the large molecular weight of the protein may potentially limit its effects on local RAAS (Gheblawi et al., 2020). rhACE2 has already undergone phase 1 and 2 clinical trials in healthy volunteers and in a small cohort of patients with ARDS (Khan et al., 2017). Moreover, it has been demonstrated that rhACE2 can significantly block the early stages of SARS-CoV-2 infections in engineered human blood vessel organoids and human kidney organoids (Monteil et al., 2020). In this context, Procko (2020) was able to engineer hACE2 sequences to obtain soluble receptors able to sequester SARS-CoV-2 RBD and inhibit its cell attachment. Remarkably, combinatorial mutants enhanced ACE2 binding to SARS-CoV-2 RBD by an order of magnitude, as compared to the wild type receptor form, and targeted ACE2 mutations might provide further improvement.

Additionally, the availability of ACE2 nanoparticles applied to nose filters, chewing gums, clothes, filters and gloves could be of help in sequestering the virus thus preventing its entry into the host (Aydemir and Ulusu, 2020). Prevention virus transmission could represent a more convenient strategy than therapeutic interventions on viral infection, avoiding interference with ACE2 and disturbance of the finely regulated RAAS axis (Aydemir and Ulusu, 2020).

### ACE2 and Other Diseases

ACE2 is a multiform protein (Feng et al., 2010, 2011). As discussed above (section Structure of ACE2) its C-terminal domain is similar to collectrin, a kidney protein involved in amino acids trafficking and insulin secretion (Kuba et al., 2013). Alterations in ACE2 have been demonstrated in Hartnup’s disease due to a disturbance in amino acids homeostasis. Indeed, ACE2 has been proposed to modulate amino acid transport in bowel and gut microbiome (Hashimoto et al., 2012).

Moreover, ACE2 participates in the regulation of metabolism, particularly of glucose homeostasis. In the pancreas, activation of the ACE2-Ang1-7-MasR pathway improves insulin secretion (Yuan et al., 2013). Obesity and high-fat diets cause a reduction in ACE2 expression in the adipose tissue, which in turn results in increased blood pressure (Gupte et al., 2008, 2012; Carsana et al., 2020).

A role for ACE2 is emerging in Alzheimer disease, since it has been shown that ACE2 can hydrolyse Beta amyloid peptides (Zou et al., 2007, 2013).

There is also increasing evidence that the RAAS system may be implicated in cancer. ACE2 was found to inhibit cancer cell growth, metastasis, and angiogenesis in breast (Yu et al., 2016; Zhang Q. et al., 2019), pancreatic (Zhou et al., 2011), and colon cancer (Bernardi et al., 2012). Another study pointed out that hepatocellular carcinoma patients with higher levels of ACE2 had longer survival times, suggesting a positive link between ACE2 expression and better prognosis (Ye et al., 2015). Studies on human xenografts in mice clearly indicated that ACE2 inhibited tumor growth by suppressing invasion and angiogenesis in Non-Small Cell Lung Cancer (NSCLC). Remarkably, the same group demonstrated that overexpression of ACE2 promotes the expression of E-cadherin at expenses of mesenchymal markers such as vimentin, and thereby inhibits the epithelial-mesenchymal transition in NSCLC models (Feng et al., 2010; Qian et al., 2013).

The discovery that lung cancer patients that harbor COVID-19 display more severe symptoms (Liang et al., 2020) set out intensive research on the possible connection between malignancies and ACE2 expression. A thorough bioinformatics analysis of the TCGA dataset on several kinds of cancer (Chai et al., 2020) has shown that:

(1) Mutation and amplification of *ACE2* gene are frequent in cancer. Yet, hot-spot mutation sites were never observed, as *ACE2* mutations were distributed across all 18 exons.
(2) *ACE2* transcription was upregulated in six tumors: colon adenocarcinoma (COAD), kidney renal papillary cell carcinoma (KIRP), pancreatic adenocarcinoma (PAAD), rectum adenocarcinoma (READ), stomach adenocarcinoma (STAD), and lung adenocarcinoma (LUAD). Interestingly, *ACE2* transcription was unchanged in lung squamous cell carcinoma (LUSC).

(3) *ACE2* transcription was downregulated in three tumors: testicular germ cell (TGCT), thyroid carcinoma (THCA), and kidney chromophobe (KICH).
(4) Changes of *ACE2* transcription were epigenetic in nature, as both mutation and copy variation of *ACE2* did not correlate with its up- or downregulation.
(5) In most cases, changes of *ACE2* transcription strongly correlated with methylation in *ACE2* promoter. More specifically, decreased methylation levels correlated with upregulation, whereas increased methylation led to downregulation.
(6) No prognostic role of ACE2 expression on patient’s survival could be demonstrated.

These data confirm the remarkable role of methylation in determining the ACE2 expression, as described in paragraph 2.2. Interestingly, a second bioinformatics study on Oncomine and TCGA databases gave slightly different results in terms of tumor-associated ACE2 expression changes, but confirmed the role of promoter hypomethylation in KIRP and uterine corpus endometrial carcinoma (UCEC) where *ACE2* transcription was significantly upregulated (Yang et al., 2020a).

## Conclusion

From the structural point of view ACE2 is closely related to ACE, and its discovery was a by-product of ACE investigations. However, this macromolecule has soon showed peculiar functional properties and has attracted increasing attention. The expression of ACE2 in the heart, kidney and vascular system pointed to a role in the regulation of blood pressure and cardiovascular homeostasis, possibly as a counterbalance to ACE activation, but this hypothesis now appears to be too simplistic. Additional functions of ACE2 are strongly suggested by its capability to modulate cytokine metabolism and the inflammatory reaction, as well as by its remarkable expression in the respiratory tract.

The recent COVID-19 pandemic has put ACE2 in the spotlight, since it has been identified as the initial target of the SARS-Cov and SARS-Cov2 viruses, representing the receptor responsible for cellular adhesion. Recent investigations have clarified the molecular details of the interaction of ACE2 with the spike glycoprotein present in this class of coronaviruses, and have partly unraveled the mechanism of protein cleavage and cellular entry. ACE2 density on cell surface and ACE2-modulated inflammatory reactions have been suggested to be important determinants of COVID-19 susceptibility and clinical course, while drugs interfering with ACE2 expression, availability and processing are under investigation as potential therapeutic agents. However, several crucial issues concerning the link between ACE2 and COVID-19 pathophysiology are still unclear, pointing to the need for focused experimental investigations and prospective clinical trials.

## Author Contributions

FS, GR, SS, LB, BS, and RB wrote the first draft of the manuscript and prepared the table and figures. RZ revised and integrated the text. All authors decided upon the general outline of the review.

## Conflict of Interest

The authors declare that the research was conducted in the absence of any commercial or financial relationships that could be construed as a potential conflict of interest.

## Footnotes

1 https://www.preprints.org/manuscript/202003.0295/v1
